# Disparate immunity proteins independently inactivate an antibacterial nuclease toxin

**DOI:** 10.1016/j.jbc.2026.113298

**Published:** 2026-06-25

**Authors:** Y. Vivian Liu, Jake Colautti, Youngchang Kim, John C. Whitney

**Affiliations:** 1 Department of Biochemistry and Biomedical Sciences, McMaster University, Hamilton, Ontario, Canada; 2 Michael DeGroote Institute for Infectious Disease Research, McMaster University, Hamilton, Ontario, Canada; 3 Structural Biology Center, X-ray Science Division, Advanced Photon Source, Argonne National Laboratory, Lemont, Illinois, USA; 4 David Braley Center for Antibiotic Discovery, McMaster University, Hamilton, Ontario, Canada

**Keywords:** bacterial toxin, DNA endonuclease, metalloenzyme, protein–protein interaction, X-ray crystallography

## Abstract

Bacteria secrete a diverse arsenal of protein toxins that inhibit the growth of competing species. To avoid self-intoxication, antibacterial toxins are encoded alongside cognate immunity proteins that typically bind to and occlude a toxin’s active site, thus neutralizing its activity. Multiple immunity proteins rarely target the same toxin, reflecting the strong coevolution between toxins and their immunity factors. Here, we identify and characterize an antibacterial toxin in a clinical isolate of *Pseudomonas aeruginosa*, which we term Tde5, and find that it is inhibited by two structurally distinct immunity proteins, Tdi5a and Tdi5b. Informatic analyses reveal that Tde5 is a member of the ββα-metal DNase superfamily, and accordingly, we find that this enzyme inhibits bacterial growth by nonspecifically degrading DNA. This antibacterial activity is counteracted by either Tdi5a or Tdi5b, suggesting that these two immunity proteins function independently to protect bacteria against Tde5-mediated toxicity. Using biochemical and biophysical approaches, we show that Tdi5a and Tdi5b interact with Tde5 with sub-nanomolar affinities and that a trimeric complex forms between these three proteins, indicating that the two immunity factors bind to the toxin through nonoverlapping interfaces. A 1.73 Å X-ray crystal structure of the Tde5-Tdi5a complex reveals that Tdi5a binds to Tde5 through a large electrostatic interface and occludes the toxin’s active site, whereas structural modeling reveals that Tdi5b binds an exosite distal to the toxin’s active site. Together, these findings define a previously unrecognized dual-immunity mechanism and expand our understanding of antibacterial toxin-immunity dynamics, informing future studies of interbacterial competition and virulence.

Bacteria secrete protein toxins to inhibit the growth of competing bacteria, to defend against predatory eukaryotic cells, and to perturb the physiology of host cells during infection (1, 2). Perhaps owing to the ubiquity of competition in microbial life, bacteria have evolved a diverse range of specialized pathways that deliver antibacterial toxins to competing cells (3, 4, 5, 6, 7, 8, 9). Despite this diversity of delivery systems, the protein toxins that are exchanged between bacteria often share a common modular architecture comprising at least two functional domains: an N-terminal trafficking domain that mediates transport between the producing and target cell, and a C-terminal domain that exerts a lethal activity (10). These C-terminal toxin domains often function as enzymes that modify or degrade physiologically essential substrates and thus disrupt homeostasis in the target cell (11, 12, 13, 14, 15, 16).

Because antibacterial toxins generally target highly conserved essential processes, they are invariably encoded alongside cognate immunity proteins that protect the producing organism and its kin from their toxic activity. These immunity proteins typically bind to the active site of enzymatic toxins with extremely high affinity, thus functioning as potent inhibitors of these deadly enzymes (17, 18, 19, 20). In most cases, antibacterial toxins are inactivated by a single highly specific cognate immunity protein. However, in some instances, multiple immunity proteins encoded within the same locus are thought to protect the producing organism against a wide range of related toxins that might be deployed by its competitors. Often, these poly-immunity loci encode multiple paralogs of the same immunity protein (10). In several characterized instances of this gene organization, one immunity paralog inactivates the cognate, adjacently-encoded toxin, while the others in the locus are thought to inactivate homologous toxins that act by a shared mechanism (21, 22, 23). In addition, some poly-immunity loci encode enzymatic immunity proteins that catalytically reverse a toxin’s activity (24, 25). Unlike canonical immunity proteins that neutralize toxins through tight binding interactions, enzymatic immunity proteins do not rely on specific protein–protein interactions. Consequently, they are thought to confer very broad protection against a large family of toxins that act by a common mechanism and are less prone to escape by adaptive coevolution (24). However, many toxin families act by mechanisms that could not conceivably be reversed by an enzymatic immunity protein, such as rapid and nonspecific degradation of nucleic acids, cell envelope peptidoglycan, or energy carrying molecules such as ATP or NAD (11, 12, 13, 26, 27). Therefore, while enzymatic reversal represents one strategy by which immunity proteins protect bacteria against antibacterial toxins, active site occlusion appears to prevail in most toxin-immunity interactions.

Here, we identify a type VI secretion system (T6SS)-associated antibacterial toxin in *Pseudomonas aeruginosa* whose activity is inhibited by two unique cognate immunity proteins. Sequence and structural analyses reveal that this toxin, which we term Tde5, belongs to the ββα-metal (ββα-Me) superfamily of DNases, and we demonstrate that it acts by nonspecifically degrading the bacterial chromosome. Strikingly, despite lacking sequence or predicted structural similarity to each other, two cognate immunity proteins, Tdi5a and Tdi5b, independently protect bacteria against the antibacterial activity of Tde5. We further demonstrate that these immunity proteins inactivate Tde5 by binding to distinct sites on this toxin, thus inhibiting its DNase activity. A 1.73 Å crystal structure of the Tde5-Tdi5a complex offers an atomic view of the binding interface between these proteins and reveals that, like many cognate immunity proteins, Tdi5a physically occludes the Tde5 catalytic site. Structural modeling further predicts that Tdi5b binds an exosite distal to the active site, suggesting a distinct mechanism of inhibition. Bioinformatics analyses reveal that this dual immunity architecture is a unique feature of closely related *Pseudomonas* strains and identify homologs of Tdi5b in gene clusters not associated with interbacterial antagonism, suggesting that this protein fold may have been recently co-opted to protect against a nuclease toxin. Our findings shed new light on the evolutionary flexibility of toxin-immunity systems and suggest that the current understanding of poly-immunity loci may not fully capture their varied functions.

## Results

### An antibacterial toxin is associated with the H4-T6SS of *P. aeruginosa*

**Figure 1:**
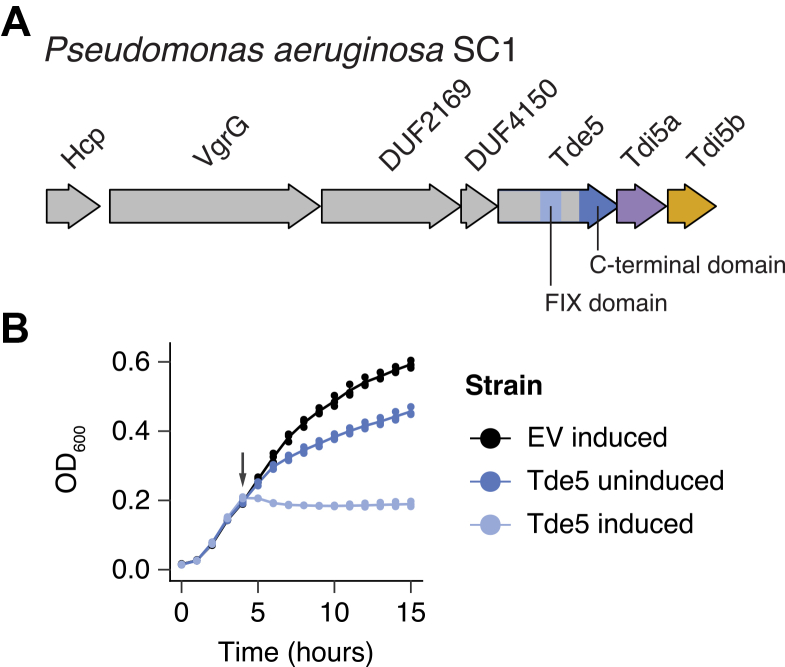
**An antibacterial toxin is associated with the H4-T6SS of *P. aeruginosa*.***A*, schematic representation of the Tde5-encoding gene cluster in *P. aeruginosa* strain SC1 (GCF_003973885.1). *B*, growth of *E. coli* harboring empty vector (EV) or plasmid encoding *tde5*. The *arrow* denotes the time at which the inducer was added. n = 3 biological replicates. T6SS, type VI secretion system.

The T6SS is a molecular machine used by a wide range of Gram-negative bacteria to deliver toxic proteins into nearby cells (28, 29). The human pathogen *P. aeruginosa* has long been known to encode three evolutionarily and functionally divergent T6SSs, termed the H1-, H2-, and H3-T6SSs, that are each thought to contribute to distinct facets of this organism’s lifestyle (30, 31, 32). A recent bioinformatic study identified an additional, so-called H4-T6SS encoded by a small subset of *P. aeruginosa* isolates (33). Two putative effectors are genetically associated with the H4-T6SS; a membrane-depolarizing toxin belonging to the VasX family, and a putative nuclease (34). Although the putative nuclease effector is encoded in a canonical effector locus alongside proteins involved in T6SS toxin recruitment (Fig. 1*A*), the mechanism underlying its potentially toxic activity and how this activity may be inhibited in effector-producing cells has not been experimentally characterized.

Since the putative nuclease effector contains a FIX domain (Fig. 1*A*), which is frequently present at the N-terminus of polymorphic toxins and facilitates their T6SS-dependent translocation (35, 36), we hypothesized that its C-terminal domain possesses antibacterial activity. To this end, we expressed the C-terminal domain under the control of an inducible promotor in *Escherichia coli*. Expression of this domain inhibited *E. coli* growth, consistent with our hypothesis that this protein functions as an antibacterial toxin (Fig. 1*B*).

### Tde5 is a ββα-Me DNase that displays nonspecific activity

**Figure 2:**
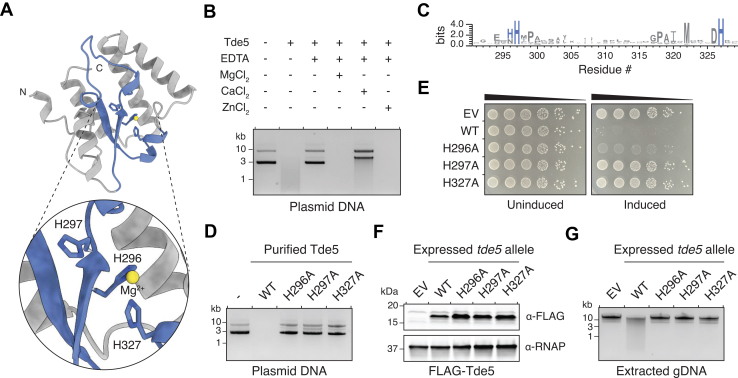
**Tde5 is a ββα-Me DNase that displays nonspecific DNase activity.***A*, AlphaFold3 model of Tde5 C-terminal domain (residues 260–394) in complex with a Mg^2+^ ion. The colored region represents the ββα motif, defined by residues 291 to 329. *B*, DNA degradation assay with pUCP20 plasmid. *C*, sequence logo representation illustrating the sequence conservation within ββα motif of Tde5. Histidine residues near the predicted metal coordination site in (*A*) are denoted in *blue*. *D*, DNA degradation assay with pUCP20 plasmid and the indicated Tde5 variants. The slower migration of plasmid DNA in the presence of Ca^2+^ reflects a known effect of this divalent cation on DNA supercoiling (78). *E*, growth of *E. coli* harboring plasmids encoding the indicated *tde5* variants in the presence or the absence of inducer. *F*, anti-His_6_ Western blot against the same samples shown in (*E*) with RNAP serving as a loading control. *G*, bacterial genomic DNA (gDNA) recovered after inducer addition from strains harbouring plasmids encoding the indicated *tde5* variations. In panels B and *D*, *E*, F, and *G*, data are representative of at least two biological replicates. ββα-Me, ββα-metal; Tde5, type VI DNase effector 5.

We next sought to determine the mechanism by which this toxin inhibits bacterial growth. Structural modeling of the C-terminal toxin domain by AlphaFold3 revealed that this protein is predicted to contain two antiparallel β-strands connected to an α-helix (Fig. 2*A*), a fold characteristic of the ββα-Me endonuclease family (37). Since these nucleases are known to be metal-dependent DNases with either nonspecific or sequence-specific interactions (38), we tested the activity of this purified domain *in vitro* using plasmid DNA as a model substrate (Fig. S1*A*). In doing so, we found that the toxin domain nonspecifically degrades plasmid DNA in a manner that can be inhibited by EDTA chelation of divalent cations (Fig. 2*B*). This inhibition is reversible by the addition of a molar excess of Mg^2+^ or Zn^2+^ but not by the addition of an excess of Ca^2+^ (Fig. 2*B*), which is consistent with previous studies of metal coordination by ββα-Me endonucleases (37). These observations suggest that this toxin likely inhibits bacterial growth by degrading DNA, a mechanism that has been reported for several T6SS-associated antibacterial toxins belonging to distinct nuclease families (27, 39). Accordingly, we named this toxin type VI DNase effector 5 (Tde5), to reflect its enzymatic activity and to maintain consistency with existing nomenclature conventions for T6SS nuclease toxins (27). For the purposes of the current study, Tde5 is hereafter used to refer to our characterization of the C-terminal toxin domain.

Having established that Tde5 degrades DNA *in vitro*, we next sought to determine whether this enzymatic activity underlies its antibacterial properties. To this end, we aimed to identify the amino acid residues responsible for its catalytic activity, reasoning that substitutions in such residues should abrogate DNase activity *in vitro* and *tde5*-dependent growth inhibition in our heterologous *E. coli* expression system. Revisiting our structural model, we identified two histidine residues predicted to coordinate a metal ion, along with a third histidine positioned adjacent to the putative metal-binding site (Fig. 2*A*). Multiple sequence alignments of Tde5 homologs revealed that all three histidine residues are highly conserved (Fig. 2*C*), suggesting that they likely contribute to metal binding or water activation, consistent with the established catalytic mechanism of ββα-Me endonucleases (37). Indeed, substitution of any of the three histidine residues with alanine abrogated Tde5-dependent degradation of purified plasmid DNA *in vitro*, demonstrating that these residues are required for the DNase activity of this enzyme (Figs. 2*D* and S1*A*). Expression of *tde5* alleles harboring these alanine substitutions abrogated *tde5*-dependent growth inhibition in *E. coli* but did not influence toxin expression, suggesting that DNase activity is responsible for the antibacterial properties of this toxin (Fig. 2, *E* and *F*). To determine whether this activity observed using plasmid DNA *in vitro* extends to chromosomal DNA *in vivo*, we next examined the integrity of genomic DNA extracted from *E. coli* cultures expressing *tde5*. Genomic DNA extracted from *tde5*-expressing cultures displayed extensive degradation, whereas DNA from cultures expressing *tde5* alanine substitution mutants remained intact (Fig. 2*G*). Together, these findings indicate that this toxin degrades the bacterial genome in our heterologous expression system and suggest that its antibacterial properties arise from nonspecific DNase activity.

### Two immunity proteins independently protect against Tde5 toxicity by binding to distinct toxin surfaces

**Figure 3:**
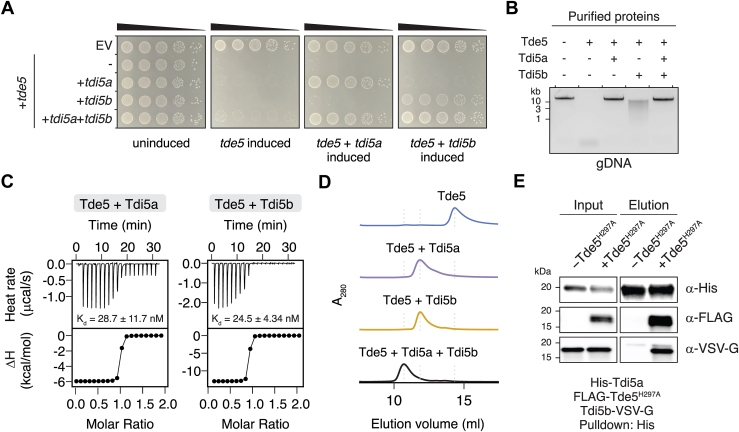
**Two immunity proteins independently prevent Tde5 toxicity by binding to distinct toxin surfaces.***A*, growth of *E. coli* harboring plasmids encoding *tde5* with either or both *tdi5a* and *tdi5b* under the control of distinct promoters on solid media containing separate inducers for each gene, as indicated. *B*, DNA degradation assay with bacterial genomic DNA from *P. aeruginosa* strain SC1, incubated with the indicated combinations of purified proteins. *C*, ITC binding analysis of the titration of Tde5 into Tdi5a (*left*) and Tdi5b into Tde5 (*right*). *Top panel* depicts the raw heats of injection whereas the *bottom panel* shows the baseline corrected integration of these heats. The interactions were fit using a single-site binding model, with the calculated binding affinity (K_d_) indicated. *D*, size exclusion chromatograms of indicated combinations of recombinant proteins. *Dotted lines* align each elution peak across samples for ease of comparison. *E*, Western blot analysis of copurification experiments between the indicated heterologously expressed tagged proteins. The Tde5^H297A^ variant was used to mitigate toxicity during protein overexpression in *E. coli*. In panels *A*, *B*, and *E*, data are representative of at least two biological replicates. Tdi5b, T6SS DNase immunity 5b; Tde5, type VI DNase effector 5; Tdi5a, T6SS DNase immunity 5a.

We next examined the mechanisms by which Tde5-producing cells protect themselves against Tde5 toxicity. Two small open reading frames are encoded downstream of Tde5, one or both of which likely confer immunity to this toxin based on genomic proximity (Fig. 1*A*). To test this hypothesis, we constructed *E. coli* strains coexpressing *tde5* with either or both putative immunity genes under the control of distinct inducible promotors and evaluated the growth of these strains under different induction conditions. Surprisingly, unlike most toxin-immunity pairs where the toxin is only inhibited by the immunity encoded immediately downstream, expression of each candidate immunity gene protected cells from Tde5-dependent growth inhibition, suggesting that these two proteins may be independently capable of inhibiting Tde5 (Fig. 3*A*). To explore this possibility directly, we purified each immunity protein (Fig. S1*A*) and tested Tde5 inhibition *in vitro*. Consistent with our results in *E. coli*, either immunity protein inhibits Tde5 DNase activity *in vitro*, albeit to differing extents (Fig. 3*B*). To reflect this activity, we named these proteins T6SS DNase immunity 5a (Tdi5a) and Tdi5b.

Given that Tdi5a and Tdi5b do not share obvious sequence or predicted structural similarity and are therefore unlikely to inhibit Tde5 through identical mechanisms, we next sought to determine how each protein inactivates Tde5. Because Tde5 nonspecifically degrades DNA and repairing a degraded haploid genome without a template would be difficult, it seems unlikely that either immunity protein would act enzymatically to reverse the DNase activity of Tde5. Therefore, we hypothesized that both immunity proteins physically interact with this toxin to inhibit its toxin activity. If so, the differences in Tde5 inhibition observed in our *in vitro* DNase assay could be explained by differences in binding sites or binding affinities between Tde5 and these immunity proteins. To begin examining this possibility, we measured the binding affinity between Tde5 and each immunity protein using isothermal titration calorimetry. Remarkably, despite their sequence and predicted structural divergence, both immunity proteins bind to Tde5 with 1:1 stoichiometry and nearly equivalent affinities in the low nanomolar range (Figs. 3*C*, S2*A*, and Table S1). This observation is consistent with our finding that Tdi5a and Tdi5b both protect *E. coli* from the antibacterial effects of Tde5 and aligns with the established paradigm that immunity proteins bind to their associated toxins with extremely high affinity (40). However, it does not explain the incomplete Tde5 inhibition by Tdi5b that we observed *in vitro* and suggests that this apparent difference may be explained by these proteins binding to different sites on Tde5.

To test whether Tdi5a and Tdi5b interact with different sites on Tde5, we subjected these proteins to size exclusion chromatography, reasoning that the formation of a trimeric complex containing all three proteins would imply that the immunity proteins must bind distinct surfaces of the toxin. As expected, Tdi5a and Tdi5b each independently interact with Tde5 (Figs. 3*D* and S2*B*). However, we could also detect the formation of a trimeric complex containing all three proteins, suggesting that both immunity proteins interact with the toxin simultaneously and therefore do not compete for the same binding site (Figs. 3*D* and S2*C*).

To independently validate this model, we also performed copurification experiments in *E. coli* using nickel affinity chromatography. By placing the affinity tag on Tdi5a, we reasoned that copurification of Tdi5b would occur only if mediated by Tde5. Consistent with our previous results, His_6_-tagged Tdi5a copurified with both Tde5 and Tdi5b (Fig. 3*E*). However, Tdi5a did not copurify with Tdi5b in the absence of the toxin, confirming that these two immunity proteins do not directly interact with each other and that the ternary complex detected in our previous experiments arises from independent interactions between each immunity protein and Tde5 (Fig. 3*E*). We therefore conclude that both immunity proteins can simultaneously interact with Tde5, likely through nonoverlapping interfaces.

### The X-ray crystal structure of Tde5-Tdi5a complex

**Figure 4:**
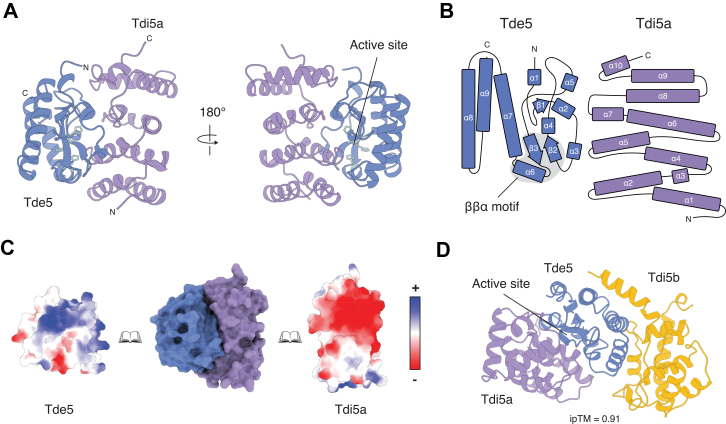
**Structural analysis of the interactions between the toxin domain of Tde5 and its cognate immunity proteins.***A*, the overall 1.73 Å structure of Tde5-Tdi5a complex, with the location of the toxin active site indicated. Conserved active site histidine residues identified in Figure 2 are colored *light blue*. *B*, schematic representation of the secondary structure of Tde5 and Tdi5a. α-helices are denoted by *tubes*, and β-strands are represented by *arrows*. The ββα motif is highlighted in *gray*. *C*, electrostatic surface representation of the Tde5-Tdi5a binding interface (Tde5 in *blue* and Tdi5a in *purple*). *D*, AlphaFold3 model of Tde5-Tdi5a-Tdi5b complex, with the active site location indicated. ββα-Me, ββα-metal; Tde5, type VI DNase effector 5; Tdi5a, T6SS DNase immunity 5a; Tdi5b, T6SS DNase immunity 5b.

To better understand the molecular contacts between Tde5 and its cognate immunity proteins, we sought to determine X-ray crystal structures of these complexes. We determined the structure of the Tde5-Tdi5a complex to a resolution of 1.73 Å (Fig. 4*A*, Table 1). The Tde5 C-terminal domain adopts a mixed α/β fold, with β2, β3, and α6 forming the conserved ββα motif characteristic of this toxin family (Fig. 4*B*). In contrast, Tdi5a adopts an entirely α-helical fold composed of pairs of antiparallel helices that resemble HEAT-like repeats (Fig. 4*B*), which are often implicated in protein–protein interactions (41). Helices α2, α5, α7, and α9 form the binding interface with Tde5 (Fig. 4*B*).

**Table 1:** X-ray data collection and refinement statistics

	Tde5 (260–393)-Tdi5a
Data collection	
Space group	*P42*_*1*_*2*
Unit cell parameters (Å; °)	*a* = *b* = 124.64, *c* = 55.04; *α* = *β* = *γ* = 90.0
Resolution range (Å)	62.32–1.73 (1.76–1.73)^a^
No. of reflections	45,823 (2471)^a^
*R*_merge_^b^	0.159 (1.501)
Completeness (%)	100 (100)
CC_1/2_^c^	0.995 (0.730)
⟨*I*/*σ*(*I*)⟩	12.3 (1.7)
Multiplicity	17.1 (16.9)
Wilson *B* factor	26.14
Structure determination	
MR initial model (PDB ID)	AF3
Refinement	
Resolution range (Å)	62.32–1.73 (1.73–1.77)
Completeness (%)	99.87 (82.1)
No. of reflections	45,734 (2670)
*R*_work_/*R*_free_^d^	0.172/0.205 (0.317/0.360)
Protein chains/atoms	2/2815
Water/Others	338/39
Mean temperature factor (Å^2^)	31.5
Protein/water/others	30.2/38.2/52.9
Coordinate deviations	
R.m.s.d. bonds (Å)/angles (°)	0.016/1.283
Clash score	3.86
Rotamer outlier (%)	0.76
Ramachandran plot^e^	
Favored (%)	99.65
Allowed (%)	0.35
Outside allowed (%)	0.00
PDB Accession Code	12FS

a Values in parentheses correspond to the highest resolution shell.

b R_merge_ = Σ*h*Σ*j*|*Ihj*–<*Ih*>|/Σ*h*Σ*jIhj*, where *Ihj* is the intensity of observation *j* of reflection *h*.

c As defined by Karplus and Diederichs (79).

d R_work_ = Σ*h*|*F*_*o*_|–|*F*_*c*_|/Σ*h*|*F*_*o*_| for all reflections, where *F*_*o*_ and *F*_*c*_ are observed and calculated structure factors, respectively. R_free_ is calculated analogously for the test reflections, randomly selected and excluded from the refinement.

e As defined by MolProbity (80).

Our cocrystal structure reveals that Tdi5a binds directly to the active site of Tde5, with a buried interaction surface of 1338 Å^2^ (Fig. 4*C*). This interface encompasses a large positively charged patch on the surface of Tde5, which likely contributes to the recognition of the anionic DNA backbone by this enzyme (Fig. 4*C*). Tdi5a binds to this site on Tde5 through a highly negatively charged surface (Fig. 4*C*), suggesting that the Tde5–Tdi5a interaction is likely mediated by strong electrostatic complementarity. Together with our biophysical data, these structural features provide an explanation as to how Tdi5a potently inhibits Tde5; by binding to the toxin through a large electrostatic interface and occluding its active site, Tdi5a interferes with Tde5 DNase activity and protects bacteria against its toxic effects. Furthermore, the finding that Tdi5a binds at the Tde5 catalytic site, together with our previous findings that both immunity proteins can simultaneously bind to this toxin, implies that Tdi5b must bind elsewhere on the toxin surface.

Despite extensive efforts, we were unable to crystallize a complex containing Tdi5b; however, AlphaFold3 confidently predicts a trimer formed between the toxin and both immunity proteins, which recapitulates many aspects of our Tde5-Tdi5a crystal structure. In this model, Tdi5a binds the Tde5 active site in a conformation consistent with our crystal structure, whereas Tdi5b is positioned on the opposite surface of the toxin (Figs. 4*D* and S2*D*), likely inhibiting Tde5 through either allosteric effects or steric hindrance of substrate binding. This arrangement agrees with our biochemical and structural data and provides an explanation as to how these two immunity proteins can simultaneously interact with Tde5.

### Tdi5a is restricted to toxin-immunity context while Tdi5b is widespread

**Figure 5:**
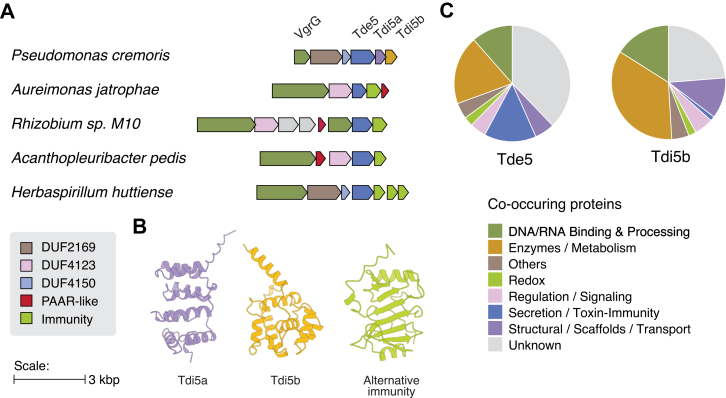
**Genomic context analyses of Tde5 and Tdi5b homologs.***A*, schematic representation of gene neighborhoods encoding Tde5 homologs in bacteria. Domains associated with polymorphic toxin systems are indicated: VgrG (valine glycine repeat protein G), PAAR (proline-alanine-alanine-arginine), PAAR-like domain (DUF4150), chaperone (DUF2169), and adapter protein (DUF4123). *B*, secondary structure comparison between Tde5-associated immunity proteins predicted by AlphaFold3. *C*, functional distribution of genes co-occurring with Tde5 and Tdi5b homologs, based on predicted function annotations. Tdi5b, T6SS DNase immunity 5b; Tde5, type VI DNase effector 5.

The unusual observation that Tde5 is inhibited by two independent immunity proteins raises the possibility that this dual immunity architecture may be a general feature of Tde5-like toxins. We therefore sought to investigate the co-occurrence of this immunity organization with Tde5-like toxins in diverse polymorphic toxin systems. To this end, we conducted a Hidden Markov Model search for Tde5 homologs and examined the genetic contexts in which these toxins are encoded. This analysis identified 139 Tde5-like effectors, many of which genetically co-occur with previously described T6SS structural components and chaperones (Fig. 5*A*) (42, 43, 44). Despite this widespread distribution of Tde5 homologs in T6SS effector loci, the dual immunity architecture appears to be a rare instance, as we were only able to identify one other case of this gene organization in a candidate T6SS effector locus of *Pseudomonas cremoris* (Fig. 5*A*). Instead, the majority of Tde5 homologs identified in our analysis co-occur with a single cognate immunity protein that is distinct from both Tdi5a and Tdi5b at a sequence and predicted structural level (Figs. 5, *A*, *B* and S3*A*). We also identified some cases in which Tde5 co-occurs with a canonical poly-immunity locus that encodes multiple paralogs of this alternative immunity protein (Figs. 5*A* and S3*A*). Interestingly, despite the structural dissimilarity between Tdi5a and the alternative immunity proteins we identified outside the genus *Pseudomonas*, AlphaFold3 confidently predicts that these immunity proteins bind to the active site interface of their associated toxins (Fig. S3*B*), suggesting that this mechanism of Tde5-like toxin inhibition has been achieved by diverse immunity protein families.

Surprisingly, our analysis revealed that Tdi5b homologs are rarely encoded alongside known components of polymorphic toxin systems (Fig. 5*C*). Instead, they are frequently found in diverse genomic neighborhoods alongside genes whose products perform a range of functions, including kinases, DNA-binding proteins, and metabolic enzymes (Fig. S4*A*). This observation suggests that these Tdi5b homologs likely function primarily in physiological contexts that are unrelated to the activation of antibacterial toxins. Together with our finding that the dual immunity organization of Tdi5a and Tdi5b is relatively uncommon, this coevolutionary pattern suggests that the Tdi5b protein fold likely arose to perform a range of cellular functions, and owing to its suitability for diverse protein–protein interactions, has been co-opted in rare instances to inactivate an antibacterial nuclease toxin.

## Discussion

Antibacterial toxins corrupt cellular homeostasis by targeting essential physiological processes or structural components of the cell. By degrading the bacterial chromosome, DNase toxins irreversibly destroy the genetic information that enables all cellular functions. Given such devastating effects on target cells, it is perhaps unsurprising that nonspecific DNases represent some of the most widely distributed toxins in nature (10, 27, 35, 39, 45, 46, 47). Here, we identify Tde5 as an antibacterial toxin belonging to the ββα-Me DNase superfamily and demonstrate that this enzyme exerts its antibacterial effects by degrading target cell DNA. We further find that Tde5 activity is inhibited by the direct binding of not one but two immunity proteins that can independently protect bacteria against DNA degradation and prevent Tde5-mediated growth inhibition. Our crystal structure of the complex between Tde5 and one of these immunity proteins, Tdi5a, together with structural modeling and biochemical evidence, strongly suggests that these immunity proteins bind distinct surfaces on Tde5. Finally, bioinformatic analyses reveal that this dual immunity organization is a unique feature of Tde5-like toxins encoded by the genus *Pseudomonas* rather than a property of Tde5 immunity in general. Together, these findings establish a unique dual immunity system in which two distinct immunity proteins separately neutralize the same toxin through independent physical contacts.

The presence of two seemingly redundant cognate immunity proteins in this system raises several questions regarding the evolutionary forces that maintain their co-occurrence. One possibility is that dual immunity may represent excess capacity that buffers the toxin-immunity interface against the otherwise severe selective constraints imposed by its essential function. In a one-to-one toxin-immunity system, minor perturbations at the binding interface that allow the toxin to escape inactivation would be purged due to the lethal consequences of unrestrained toxin activity in the producing cell. However, the presence of a second, functionally nonredundant immunity protein that binds to a distinct toxin surface may permit mutational exploration of one toxin-immunity interface and escape of the toxin from neutralization by the other immunity protein. This can facilitate the accumulation of sequence and structural variation at one toxin-immunity interface, leading to the generation of novel protein–protein interaction specificities without affecting producing cell fitness. Over time, genetic drift may render both immunity proteins essential for toxin neutralization (48), contributing to highly stringent kin discrimination during interbacterial competition, where small variations in toxin-immunity matching can alter competitive outcomes (49). The infrequent occurrence of the dual-immunity system observed in our bioinformatics analysis is perhaps unsurprising under this paradigm, as the initial excess capacity offered by redundant immunity proteins provides little to no fitness advantage and is therefore unlikely to be propagated by positive selection. An alternative explanation is that the dual immunity system reflects recombination diversification within toxin-immunity modules, where duplication and horizontal transfer allow co-opting of putative immunity proteins from other contexts and lead to the co-occurrence of multiple immunity proteins that recognize the same toxin (21, 50). Consistent with this possibility, our analysis revealed that Tde5 homologs are frequently associated with alternative immunity proteins and that Tdi5b homologs occur in diverse genomic contexts. Further bioinformatic and biochemical studies of functionally redundant but sequence diverse immunity proteins will be required to explore these possibilities.

Although both immunity proteins inhibit Tde5, the mechanism of neutralization by Tdi5b remains unclear. In contrast with Tdi5a, which binds to Tde5 and occludes its active site, the mode by which Tdi5b attenuates toxicity remains to be determined. One possibility is that Tdi5b induces global conformational changes that allosterically disrupt the catalytic site, a mechanism that has been described previously (51). However, Tdi5a could still bind the Tde5-Tdi5b complex in a trimeric assembly, suggesting the active site remains structurally accessible and that any Tde5 conformational changes induced by Tdi5b must be small enough to preserve the Tdi5a binding interface. Alternatively, Tdi5b may inhibit Tde5 through steric hindrance, preventing substrate access without directly engaging the active site. This seems to be an attractive possibility given that nucleic acid substrates are large polymers that occupy surfaces beyond the active site when bound to nucleases (52). A similar strategy has been described for antibacterial colicins E3 and E9, whose immunity proteins bind exosites distal to their active sites (20, 53). Steric hindrance by Tdi5b could also explain the seemingly imperfect Tde5 inhibition that we observed *in vitro*, as this mode of inhibition may be less effective than direct active site occlusion by Tdi5a. Alternatively, the incomplete inhibition we observed might be explained by differences in the dissociation rate constant (k_off_), as transient dissociation between Tde5 and Tdi5b could periodically allow substrate binding and degradation, permitting limited DNA cleavage by Tde5 even in the presence of excess Tdi5b. In summary, future structural and biochemical studies will be needed to conclusively inform the mechanism by which Tdi5b inhibits Tde5.

The Tde5 system represents a unique configuration among toxin-immunity pairs. Although previous studies have described T6SS-associated toxins that are neutralized by either direct orthosteric or allosteric inhibition, the coexistence of two immunity proteins simultaneously targeting different toxin surfaces has not been observed (17, 18, 19, 51). Furthermore, both immunity proteins inhibit the toxin through direct physical interaction rather than through a combination of binding and enzymatic detoxification (24, 25). Finally, in many systems, expanded protection against related toxins is achieved through orphan immunity proteins encoded distally in the genome, which provide cross-protection independent of the cognate toxin locus (50, 54). In contrast, both Tde5 immunity proteins are encoded immediately downstream of the toxin gene, indicating the dual protection is genetically coupled to a single effector rather than distributed across the genome. Together, these features define a distinct dual-immunity mechanism that expands the known diversity of toxin-immunity pairs in bacterial competition.

## Experimental procedures

### Bacterial strains and growth conditions

A full list of strains used in this study can be found in Table S2. *E. coli* strain XL1 Blue (Novagen) was used for plasmid maintenance and toxicity experiments, and strain BL21 (DE3) pLysS (Novagen) was used for recombinant protein expression. *E. coli* strains were grown in lysogeny broth (LB) medium (10 g/L tryptone, 5 g/L yeast extract, 10 g/L NaCl) at 37 °C shaking at 220 RPM. When appropriate, *E. coli* cultures were supplemented with 50 μg/ml kanamycin, 100 μg/ml ampicillin, 25 μg/ml chloramphenicol, 30 μg/ml gentamicin, 200 μg/ml trimethoprim, 0.1% L-rhamnose, 0.1% L-arabinose, and 1 mM IPTG.

### DNA manipulation and plasmid construction

A full list of plasmids and oligonucleotides used in this study can be found in Tables S3 and S4, respectively. All expression vectors were constructed using standard restriction enzyme-based cloning procedures (55). Primers were synthesized and purified by Integrated DNA Technologies. Phusion polymerase, restriction enzymes, and T4 DNA ligase were obtained from New England Biolabs. Sanger sequencing was performed by The Centre for Applied Genomics. Whole plasmid sequencing was performed by Plasmidsaurus using Oxford Nanopore Technology with custom analysis and annotation.

### Sequence alignment and sequence logo generation

Homologous sequences to the Tde5 toxin domain were identified using iterative searches with JackHMMER (HMMER v3.4) against the UniProtKB database (2025_01) (56, 57). Searches were performed for three iterations using an inclusion E-value threshold of 0.005. Full-length sequences of significant hits were retrieved, clustered using MMseqs2 at 95% sequence identity and 100% coverage to remove highly similar sequences (58), filtered by length to exclude large multidomain proteins, and filtered using a domain E-value cutoff of 1e-5 to remove spurious matches, yielding 178 nonredundant candidates. Multiple sequence alignment was generated using ClustalO with default parameters (59), and columns not corresponding to the original toxin domain were removed. The resulting alignment was visualized using WebLogo (v.3.9.0) (60).

### Bacterial growth curves

Growth curves were conducted in liquid culture shaking at 220 RPM at 37 °C. Overnight cultures of *E. coli* XL1 Blue harbouring the indicated plasmids were diluted to an OD600 of 0.05 in 200 μl LB supplemented with appropriate antibiotics in a 96-well microtiter plate. The plate was shaken at 37 °C in a BioTek Synergy H1 multimode reader (Agilent), and the absorbance at 600 nm was measured every hour. After 4 h of incubation, L-rhamnose was added to a final concentration of 0.1% (w/v), and the plate was incubated for a further 11 h, with absorbance measurements every hour.

### Bacterial toxicity assays

Cultures were grown overnight in 3 ml LB supplemented with appropriate antibiotics at 37 °C shaking at 220 RPM. Overnight cultures were normalized to an OD600 of 1.0 and were diluted in 10-fold series in a 96 well microtiter plate. A total of 5 μl of 10^−1^-10^−5^ dilutions were spotted onto LB 1.5% (w/v) agar containing 0.1% (w/v) L-rhamnose to induce toxin expression from pSCrhaB2-CV-derived vectors, and/or 0.1% (w/v) L-arabinose or 1 mM IPTG to induce immunity expression from pBAD33-or pPSV39-derived vectors, respectively. Plates were incubated at 37 °C and imaged after 18 h.

To enhance detection of WT Tde5 in Western blot, epitope-tagged toxin variants were expressed in an immunity background. Specifically, *tde5* alleles were ligated into the rhamnose-inducible vector pSCrhaB2-CV and transformed into an *E. coli* XL1 Blue strain expressing the immunity protein Tdi5a from plasmid pBAD33.

### Recombinant protein expression and purification

For purification of the Tde5 C-terminal toxin domain and its point mutants, all variants of the effector were co-expressed alongside the Tdi5a immunity protein on pETDuet, to prevent cellular intoxication. An on-column denaturation and refolding strategy was then used to remove the immunity protein, allowing for the purification of the N-terminal His_6_-tagged effector. C-terminal His_6_-tagged immunity proteins were expressed and purified separately using pET29b. *E. coli* BL21 (DE3) pLysS (Novagen) strains harboring expression vectors were grown in LB at 37 °C shaking at 220 RPM overnight with appropriate antibiotics. A total of 20 ml of starter culture was used to inoculate each 1 L LB, which was allowed to grow at 37 °C shaking at 220 RPM to an OD600 of 0.6 to 0.8. IPTG was added at a final concentration of 1 mM to induce protein expression. The culture was subsequently incubated for 16 h at 18 °C shaking at 220 RPM before cells were harvested by centrifugation at 6000*g* for 18 min. Cells were resuspended in lysis buffer (300 mM NaCl, 50 mM Hepes NaOH pH 7.5) and lysed by sonication (30% amplitude, 15 s pulses for 4.5 min). Lysates were clarified by centrifugation at 16,000 *g* for 45 min at 4 °C and loaded onto a 1 ml gravity flow Ni-NTA agarose (Qiagen) column preequilibrated with 10 ml wash buffer (300 mM NaCl, 50 mM Hepes NaOH pH 7.5, 10 mM imidazole). For assays requiring free Tde5, the Tde5-Tdi5a complex was denatured with 6 M guanidine HCl supplemented in the lysis buffer to remove untagged immunity, then refolded on the NTA column by linear gradient reduction of guanidine concentration (4 M, 2 M). The column was subsequently washed 3 times with 30 ml wash buffer and His_6_-tagged proteins were eluted by the addition of 4 ml elution buffer (300 mM NaCl, 50 mM Hepes NaOH pH 7.5, 400 mM imidazole). Eluted proteins were further purified by gel filtration on a HiLoad 16/600 Superdex 200 size exclusion chromatography column (GE Healthcare) equilibrated with SEC buffer (20 mM Hepes NaOH pH 7.5, 150 mM NaCl). Protein purity was analyzed by SDS-PAGE stained with Coomassie Brilliant Blue R250. Protein concentration was measured using a NanoDrop instrument (Thermo Fisher Scientific), and proteins were concentrated to 50 μM using a 10 kDa molecular weight cutoff centrifugal filter device (MilliporeSigma). Proteins were used immediately or snap frozen in liquid nitrogen and stored at −80 °C for later use.

### Protein SDS-PAGE and Western blotting

Analysis of proteins by SDS-PAGE was performed using homemade 10 cm Tris-glycine SDS-PAGE gels containing 16% (w/v) acrylamide. Samples were boiled in the presence of 1 x SDS PAGE loading dye at 95 °C for 10 min, centrifuged at 21,000 *g* for 1 min, and loaded onto a SDS-PAGE gel. Gels were run first for 10 min at 95 V, followed by 45 min at 195 V. To visualize total proteins, gels were subsequently stained with Coomassie Brilliant Blue for 30 min at RT with gentle rotation and destained with 30% (v/v) methanol and 10% glacial acetic acid (v/v).

For Western blotting, total protein was wet transferred from the SDS gel onto a nitrocellulose membrane (BioRad) using the Mini Trans-Blot electrophoresis system (BioRad). The transfer was run at 100 V for 30 min, after which the membrane was blocked in Tris-buffered saline pH 7.2 containing 0.05% (v/v) Tween-20 (TBS-T) and 0.5% (w/v) nonfat milk (BioRad) for 30 min at room temperature with gentle shaking at 60 RPM. Primary antibodies were added (1:5000) and the membrane was incubated for 1.5 h at RT with gentle shaking. Following incubation, the membrane was washed 3× for 5 min each with 10 ml TBS-T. Secondary antibody was added diluted 1:5000 in 10 ml TBS-T and the membrane was incubated for 1 h at RT with gentle shaking. The membrane was then washed 3× for 5 min each with 10 ml TBS-T, developed by the addition of Clarity Max ECL substrate (BioRad) and visualized using a ChemiDoc instrument (BioRad).

### Isothermal titration calorimetry

ITC measurements were performed with a MicroCal PEAQ-ITC microcalorimeter (Malvern). Titrations were carried out with 1 mM of the purified titrant in the syringe and 100 μM of analyte in the cell. The titration experiment consisted of one small priming injection (0.4–2.0 μl) followed by 18 2.0 μl injections with 100 s intervals between each injection. The ITC data were analyzed using the MicroCal PEAQ-ITC Analysis software (version 1.52), fit using a single-site binding model, and corrected for baseline and offset.

### Size exclusion chromatography

Analytical size exclusion chromatography was performed using a 10/300 Gl Superdex 200 size exclusion column (GE Healthcare) on an AKTA system (GE Healthcare). Each of the indicated purified proteins was combined at a final concentration of 120 μM and loaded onto the size exclusion column preequilibrated with buffer containing 20 mM Hepes NaOH pH 7.5, 150 mM NaCl. Eluted fractions were concentrated by TCA precipitation and analyzed by SDS-PAGE, followed by staining with Coomassie Brilliant Blue R250.

### Copurification experiment

For copurification experiments, *E. coli* BL21 (DE3) pLysS strains harboring the indicated vectors were grown in 100 ml LB containing appropriate antibiotics at 37 °C, shaking at 220 RPM, to an OD600 of 0.6 to 0.8. IPTG was added at a final concentration of 1 mM to induce protein expression. The culture was subsequently incubated for 18 h at 18 °C. Cells were harvested by centrifugation at 9000 × *g*, resuspended in buffer containing 50 mM Hepes NaOH pH 7.5, 500 mM NaCl, and lysed by sonication. Lysates were clarified as above and loaded onto a 500 μl gravity flow Ni-NTA column preequilibrated with 10 ml wash buffer (50 mM Hepes NaOH pH 7.5, 500 mM NaCl, and 10 mM imidazole pH 7.5). Columns were washed four times with 10 ml wash buffer. His_6_-tagged proteins/protein complexes were eluted in 500 μl buffer containing 50 mM Hepes NaOH pH 7.5, 300 mM NaCl, and 400 mM imidazole. Proteins present in the input and eluted fractions were analyzed by Western blotting.

### Protein structure prediction

A full list of confidence metrics for all protein models used in this study can be found in Table S5. Protein structure predictions were generated with AlphaFold3 and selected based on the per-residue predicted Local Distance Difference Test (pLDDT) scores for each chain (61). Structural analysis and visualization were performed using UCSF ChimeraX (v.1.11) (62). Electrostatic surface representations and predicted alignment error plots were generated using default parameters.

### DNase activity assay

Genomic DNA isolated from *P. aeruginosa* strain SC1 using the Monarch Spin gDNA Extraction Kit and pUCP20 plasmid DNA were used as the substrates in the DNase activity assay. A total of 100 ng of DNA was added in the presence or absence of purified Tde5 or its point mutants (100 nM), immunity protein (100 nM), EDTA (100 μM), and/or divalent cation and chloride salts (1 mM) as indicated in a final volume of 10 μl. Reactions were incubated at 37 °C for 20 min and halted by the addition of DNA electrophoresis loading dye. Nucleic acids were assessed by 1% agarose electrophoresis, stained using ethidium bromide, and visualized using a ChemiDoc instrument (BioRad).

### Protein crystallization, x-ray data collection, and structural determination

Crystals were grown by the hanging drop vapor diffusion method at RT. To identify initial crystallization conditions, Tde5-Tdi5a crystallization trials were conducted using a 384-well sparse matrix screen (Microlytic). Trials were conducted in 48-well VDX plates (Hampton Research) by hand with 4 μl drops containing a 1:1 ratio of protein (10 mg/ml) and reservoir solution equilibrated over 200 μl of reservoir solution. Crystals appeared after 5 days of growth in 0.25 M calcium acetate, 0.1 M imidazole:HCl pH 8.0, 6% (w/v) PEG8000. Crystals were cryoprotected in crystallization solution supplemented with 25% (v/v) ethylene glycol prior to vitrification.

The x-ray diffraction images were recorded on the Eiger 6M detector using 0.45° rotation and 0.2 s exposure for 360° at the NE-CAT 24-IDE beamline at the Advanced Photon Source, Argonne National Laboratory. The diffraction data were collected to resolution of 1.73 Å at the wavelength of 0.97934 Å. The data were processed with P42_1_2 space group and scaled with RAPD2 (63) at the beamline. Intensities were converted to structure factor amplitudes in the Ctruncate program (64, 65) from the CCP4 package (66). The structures were determined using Molrep (67) implemented in the HKL3000 software package (68) using the AF3 (61) model as a search model. The initial structure containing one hetero-dimer in the asymmetric unit was refined as rigid bodies and then all atoms were refined by 12 cycles of REFMAC (66, 69) before they were iteratively refined using COOT (70) and PHENIX (71). Throughout the refinement, the same 5% of reflections were kept out from the refinement in both REFMAC and PHENIX refinement. The final structure converged to R_work_ = 0.172 and R_free_ = 0.205. The stereochemistry of the structures were checked with PROCHECK (72), MolProbity (73) and the Ramachandran plot and validated with the PDB validation server. The statistics of data collection and processing and the refinement are given in Table 1. The atomic coordinates and structure factors have been deposited in the Protein Data Bank under accession code 12FS.

### Genomic neighborhood bioinformatics analysis

Homologous sequences were identified using iterative searches with JackHMMER (HMMER v3.4) against the UniProtKB database (2025_01). Searches were performed for three iterations using an inclusion E-value threshold of 0.005. Full-length sequences of significant hits were retrieved, clustered using MMseqs2 at 95% sequence identity and 100% coverage to remove highly similar sequences, filtered by length to exclude large multidomain proteins, and filtered using a domain E-value cutoff of 1e-5 to remove spurious matches. Surrounding genes were visualized by EFI-GNT, excluding gene fragments during analysis (74). Gene categories were determined using Pfam clans (75), with manual correction for unknown clusters.

## Data availability

The X-ray crystallographic structure of Tde5-Tdi5a was deposited in the PDB with the ID 12FS.

## Supporting information

This article contains supporting information (76, 77).

## Conflict of interest

The authors declare that they have no conflicts of interest with the contents of this article.
