# Supplementary material for: Disparate immunity proteins independently inactivate an antibacterial nuclease toxin
Source: J Biol Chem. 2026 Jun 25;302(8):113298. doi: 10.1016/j.jbc.2026.113298 (PMC13392607; doi:10.1016/j.jbc.2026.113298)
Supplement: Supporting Figures and Tables [file mmc1.docx]

Supporting information

**Disparate immunity proteins independently inactivate an antibacterial nuclease toxin**

Y. Vivian Liu^1,2^, Jake Colautti^1,2^, Youngchang Kim^3^, John C. Whitney^1,2,4^

^1^Department of Biochemistry and Biomedical Sciences, McMaster University, Hamilton, Ontario, L8S 4K1, Canada

^2^Michael DeGroote Institute for Infectious Disease Research, McMaster University, Hamilton, Ontario, L8S 4K1, Canada

^3^Structural Biology Center, X-ray Science Division, Advanced Photon Source, Argonne National Laboratory, Lemont, Illinois, USA

^4^David Braley Center for Antibiotic Discovery, McMaster University, Hamilton, Ontario, L8S 4K1

Supplementary Figures S1-S4

Supplementary Tables S1-S5

Supplementary References

Running title: Dual immunity proteins inactivate nuclease toxin

Keywords: Bacterial toxin, DNA endonuclease, metalloenzyme, protein-protein interaction, X-ray crystallography

To whom correspondence may be addressed: John C. Whitney

Email – [jwhitney@mcmaster.ca](mailto:jwhitney@mcmaster.ca)

Telephone - +1 (905) 525-9140

**
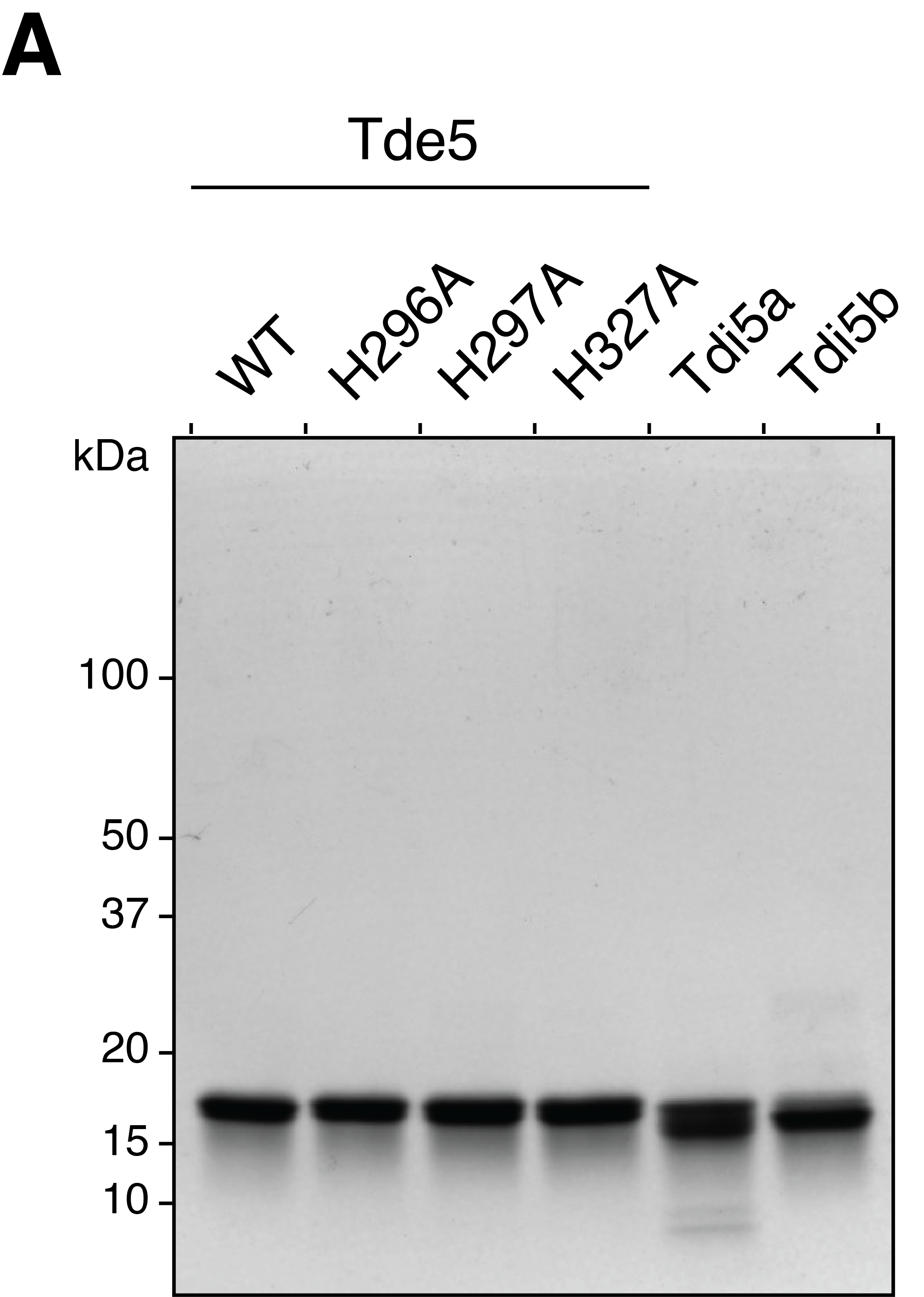
**

**Figure S1: Purified proteins used in this study.** *A,* Coomassie-stained SDS–PAGE analysis of purified proteins used in all *in vitro* assays.


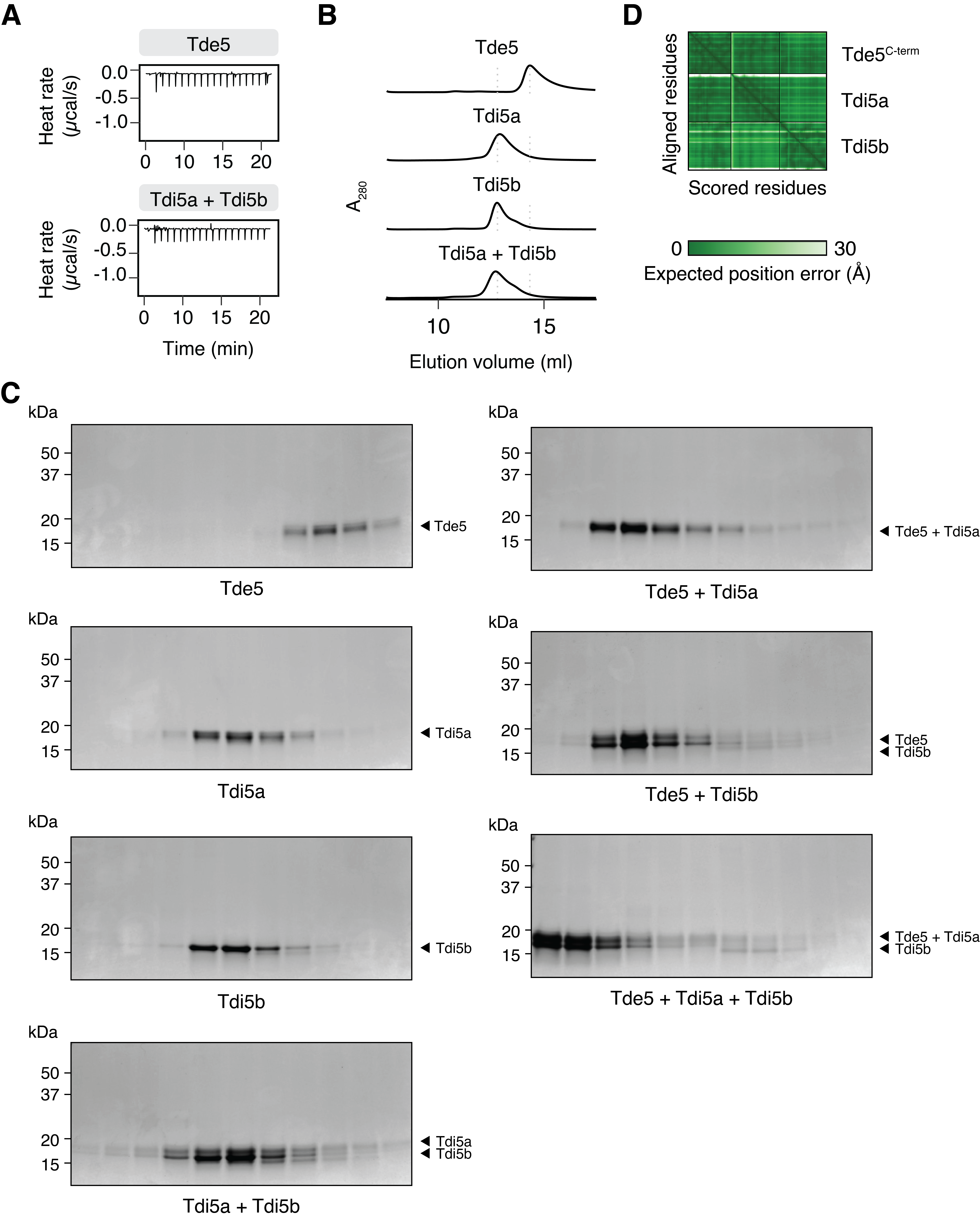


**Figure S2: Tde5 forms stable dimeric and trimeric complexes with Tdi5a and Tdi5b.** *A,* ITC binding analysis of the indicated proteins. The raw heats of injection of each titration is indicated. *B,* Size exclusion chromatograms of indicated combinations of recombinant proteins. Dotted lines align each elution peak across samples for ease of comparison. *C,* SDS-PAGE analysis of size exclusion chromatography fractions from (B) and Figure 3(D) stained with Coomassie brilliant blue. *D,* AlphaFold3-generated predicted aligned error (PAE) plot of the trimeric Tde5-Tdi5a-Tdi5b complex. Colour corresponds to expected position error as indicated by the scale bar.


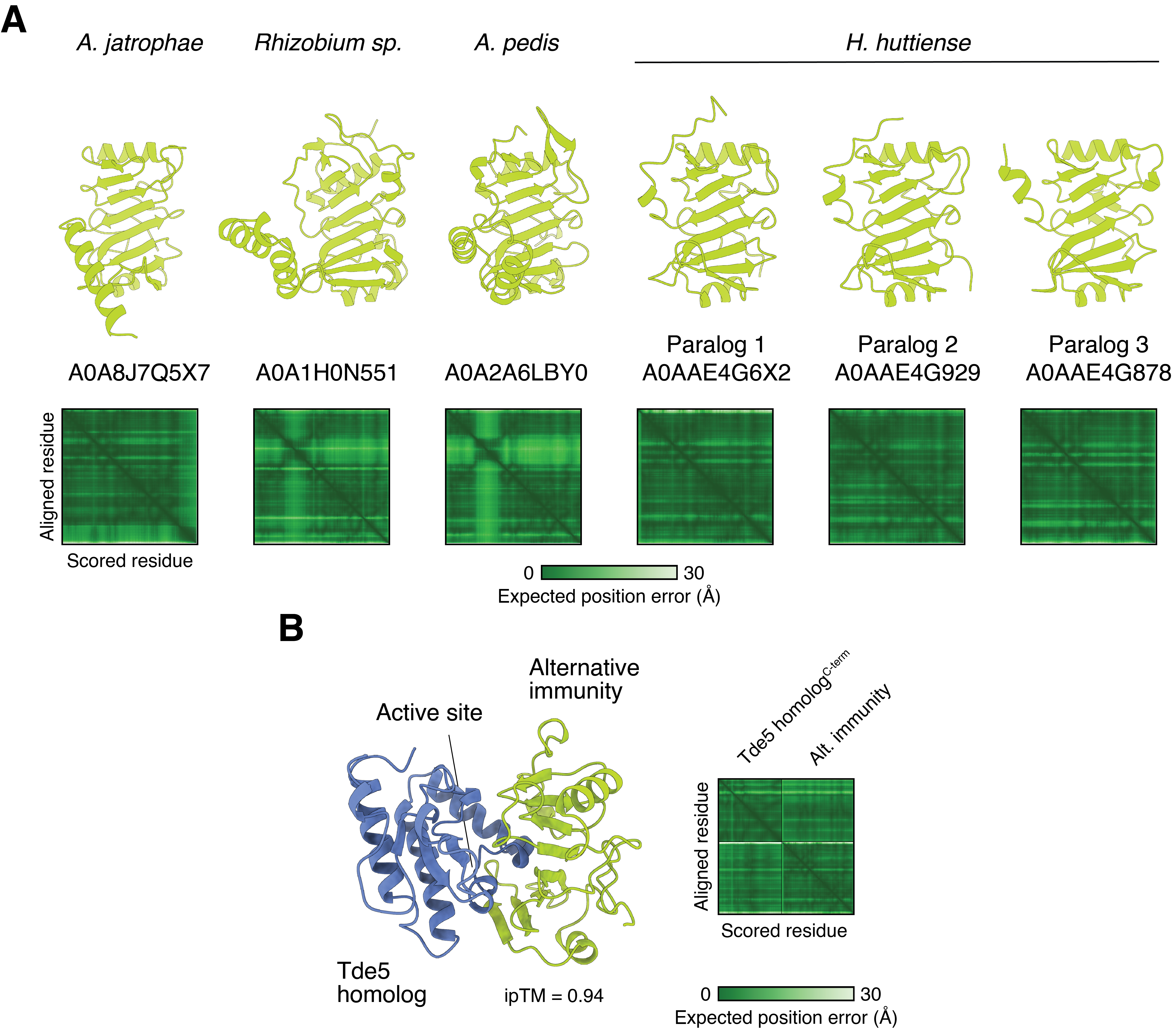


**Figure S3: Tde5 homologs are associated with alternative immunity proteins.** *A,* AlphaFold3 models and PAE plots of alternative immunity proteins identified in Figure 5(A). Paralog 1 from *H. huttiense* corresponds to the representative alternative immunity protein in 5(B). Colour corresponds to expected position error as indicated by the scale bar. *B,* AlphaFold3 model and PAE plot of a representative Tde5-alternative immunity complex. with the alternative immunity also shown in 5(B). Colour corresponds to expected position error as indicated by the scale bar.


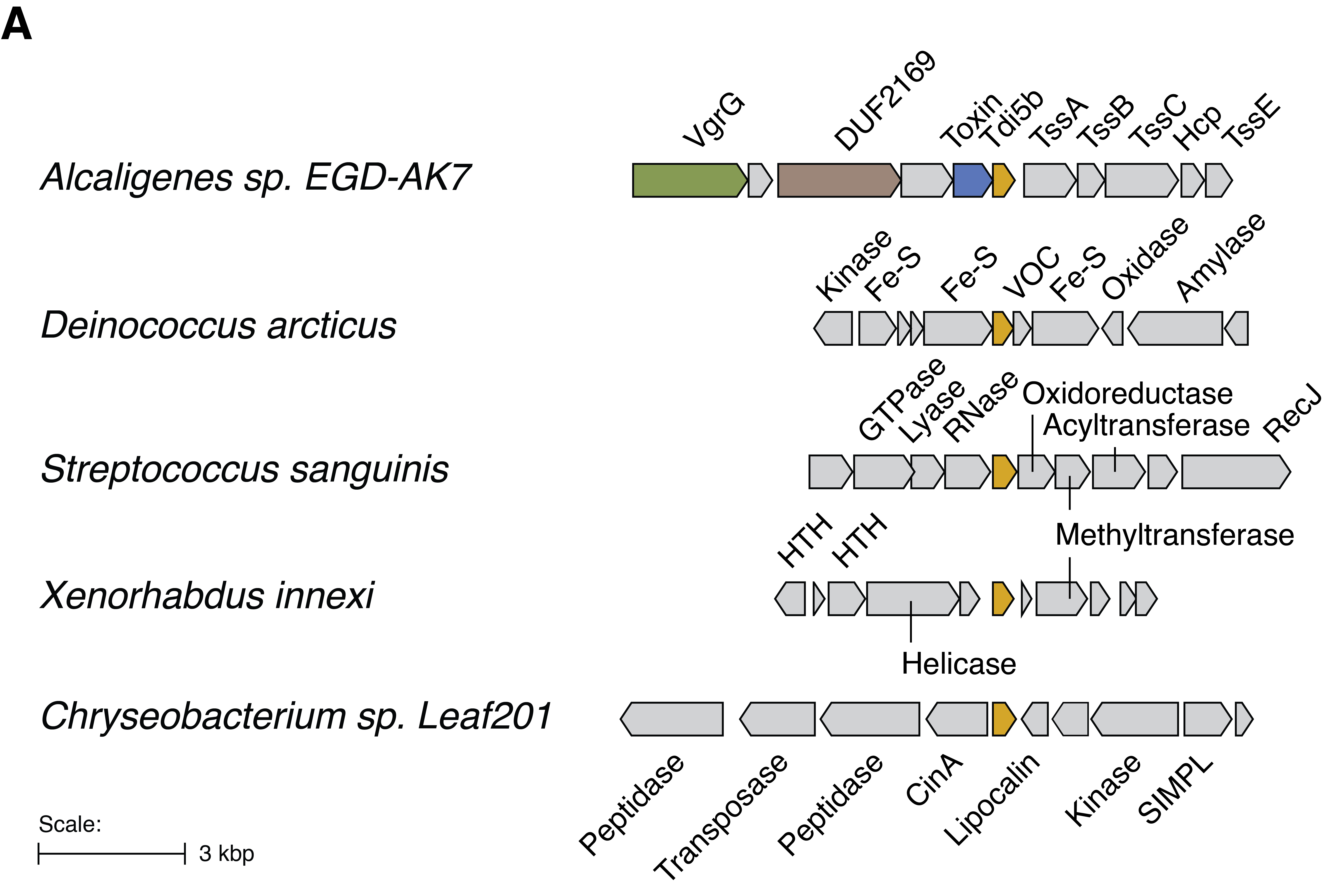


**Figure S4: Tdi5b homologs are associated with diverse genomic neighbourhoods.** *A,* Schematic representation of gene neighbourhoods encoding Tdi5b homologs in bacteria. Known domains associated with polymorphic toxin systems are coloured as in Figure 5(A).

**Table S1: Thermodynamic parameters measured by ITC**

| **Protein complex** | **K_d_ (nM)** | **N (sites)** | **ΔH  (kcal/mol)** | **TΔS  (kcal/mol)** | **ΔG (kcal/mol)** |
| --- | --- | --- | --- | --- | --- |
| Tde5-Tdi5a | 28.7 ± 11.7 | 1.0 | -6.0 | 4.3 | -10.3 |
| Tde5-Tdi5b | 24.5 ± 4.34 | 0.8 | -13.0 | -2.6 | -10.4 |

**Table S2: Strains used in this study**

| **Organism** | **Genotype** | **Description** | **Reference** |
| --- | --- | --- | --- |
| *E. coli* XL-1 Blue | *recA1 endA1 gyrA96 thi-1 hsdR17 supE44 relA1 lac* [F´ *proAB lacI^q^*ZΔ*M15* Tn*10* (Tet^R^)] | Cloning strain | Novagen |
| *E. coli* BL21 (DE3) pLysS | F- ompT gal dcm lon hsdSB(rB^-^ mB^-^) λ(DE3) pLysS(cm^R^) | Protein expression strain | Novagen |

**Table S3: Plasmids used in this study**

| **Plasmid** | **Relevant features** | **Reference** |
| --- | --- | --- |
| pSCrhaB2-CV | Expression vector with *PrhaB*, C-terminal VSV-G tag, Tmp^R^ | (76) |
| pSCrhaB2-CV::EIGOCN_17155_260-CT | Tde5 expression vector | This study |
| pSCrhaB2-CV::EIGOCN_17155_260-CT_N-FLAG | Tde5 expression vector, N-terminal FLAG tag |  |
| pSCrhaB2-CV::EIGOCN_17155_260-CT_H296A_ N-FLAG | Tde5^H296A^ expression vector, N-terminal FLAG tag | This study |
| pSCrhaB2-CV::EIGOCN_17155_260-CT_H297A_ N-FLAG | Tde5^H297A^ expression vector, N-terminal FLAG tag | This study |
| pSCrhaB2-CV::EIGOCN_17155_260-CT_H327A_ N-FLAG | Tde5^H327A^ expression vector, N-terminal FLAG tag | This study |
| pBAD33.1 | Expression vector with *araBp*, Cm^R^ | Addgene |
| pBAD33.1::EIGOCN_17150.1* | Tdi5a expression vector | This study |
| pPSV39-CV | Expression vector with *lacUV5* promoter, Gm^R^ | (77) |
| pPSV39-CV::EIGOCN_17150 | Tdi5b expression vector | This study |
| pETDuet-1 | Co-expression vector with *lacI*, T7 promoter, N-terminal His_6_ tag in MCS-1, Amp^R^ | Novagen |
| pETDuet-1::EIGOCN_17155_260-CT_N-His_6_::EIGOCN_17150.1 | Tde5/Tdi5a co-expression vector | This study |
| pETDuet-1::EIGOCN_17155_260-CT_H296A_N-His_6_::EIGOCN_17150.1 | Tde5^H296A^/Tdi5a co-expression vector | This study |
| pETDuet-1::EIGOCN_17155_260-CT_H297A_N-His_6_::EIGOCN_17150.1 | Tde5^H297A^/Tdi5a co-expression vector | This study |
| pETDuet-1_N-His_6_::EIGOCN_17155_260-CT_H327A::EIGOCN_17150.1 | Tde5^H327A^/Tdi5a co-expression vector | This study |
| pETDuet-1::EIGOCN_17150.1_N-His_6_:: EIGOCN_17155_260-CT_H297A_N-FLAG | Pull-down co-expression vector, N-terminal His_6_ tag on Tdi5a, N-terminal FLAG tag on Tde5^H297A^ | This study |
| pET29b | Expression vector with *lacI*, T7 promotor, C-terminal His_6_ tag, Kan^R^ | Novagen |
| pET29b::EIGOCN_17150.1 | Tdi5a expression vector | This study |
| pET29b::EIGOCN_17150 | Tdi5b expression vector | This study |
| PET29b::EIGOCN_17150_C-VSV-G | Pull-down expression vector, C-terminal VSV-G tag on Tdi5b | This study |

* Unannotated ORF between EIGOCN_17150 & EIGOCN_17155, with genomic interval 3,636,523 🡪 3,636,041

**Table S4: Oligonucleotides used in this study**

| **Oligonucleotide** | **Sequence (5’ to 3’)** | **Description** |
| --- | --- | --- |
| pSC_Tde5_F | CACATATGCCATGGGACCCCTGCGCAGCC | For construction of pSCrhaB2-CV:: Tde5_260-CT |
| pSC_Tde5_C-VSV-G_R | TCTGTATATCTAGATATGTAACCTTTGCATTTTGCCC |  |
| pSC_Tde5_N-FLAG _F | CACATATGCCATGGGACTACAAAGACGATGACGACAAGGACCCCTGCGCAGCC | For construction of epitope-tagged Tde5 mutants |
| pSC_Tde5_R | TCTGTATATCTAGATCATATGTAACCTTTGCATTTTGCCC |  |
| H296A_F | CAGAGAGTCTGCGCACATGCCTGCGG | For construction of pSCrhaB2-CV:: Tde5_260-CT_H296A |
| H296A_R | CCGCAGGCATGTGCGCAGACTCTCTG |  |
| H297A_F | AGAGTCTCACGCGATGCCTGCGGAC | For construction of pSCrhaB2-CV:: Tde5_260-CT_H297A |
| H297A_R | GTCCGCAGGCATCGCGTGAGACTCT |  |
| H327A_F | TAAGGCAGACGCGGAACAGACAGCC | For construction of pSCrhaB2-CV:: Tde5_260-CT_H327A |
| H327A_R | GGCTGTCTGTTCCGCGTCTGCCTTA |  |
| pBAD33_Tdi5a_F | TCGAGCTCGGTACCACGGGAGAAAGATGGGCAAAATGCAAAGGTTACATATG | For construction of pBAD33.1::Tdi5a |
| pBAD33_Tdi5a_R | AAACAGCCAAGCTTTCACTTGTCGTCATCGTCTTTGTAGTCTTTGGCGTAGTATATATCAGAAAAAGG |  |
| pPSV39_Tdi5b_F | CAATTTCAGAATTCACGGGAGAAAGATGAAGGACATCCTTAAAGATTTTCTAG | For construction of pPSV39-CV::Tdi5b |
| pPSV39_Tdi5b_R | GCCTGCAGGTCGACTCAGGTAGAGTCCAGGCCCAGGAGCGGGTTCGGGATCGGCTTACCCAGTTTTTTCCCTTCCGGCTC |  |
| Duet_NHis_Tde5_F | CAGGATCCGAATTCACCATGGGACCCCTGCG | For construction of pETDuet-1::Tde5_260-CT_N-His_6_::Tdi5a; protein purification |
| Duet_NHis_Tde5_R | GCGGCCGCAAGCTTTCATATGTAACCTTTGCATTTTGCCC |  |
| Duet_Tdi5a_F | GAGATATACATATGGGCAAAATGCAAAGGTTACACATG |  |
| Duet_Tdi5a_F | GGCCGGCCGATATCTCATTTGGCGTAGTATATATCAGAAAAAG |  |
| pET29b_Tdi5a_CHis_F | GAGATATACATATGGGCAAAATGCAAAGGTTACACATG | For construction of pET29b::Tdi5a; protein purification |
| pET29b_Tdi5a_CHis _R | TGGTGGTGCTCGAGTTTGGCGTAGTATATATCAGAAAAAGG |  |
| pET29b_Tdi5b_CHis _F | GAGATATACATATGAAGGACATCCTTAAAGATTTTCTAG | For construction of pET29b::Tdi5b; protein purification |
| pET29b_Tdi5b_CHis _R | TGGTGGTGCTCGAGCAGTTTTTTCCCTTCCGGCTC |  |
| Duet_NHis_Tdi5a_F | CAGGATCCGAATTCAGGCAAAATGCAAAGGTTACACATGATG | For construction of pETDuet-1::Tdi5a_N-His_6_::Tde5_260-CT_N-FLAG; pull-down |
| Duet_NHis_Tdi5a_R | GCGGCCGCAAGCTTTCATTTGGCGTAGTATATATCAGAAAAAG |  |
| Duet_N-FLAG_Tde5_F | GAGATATACATATGCCATGGGACTACAAAGACGATGACGACAAGGACCCCTGCGCAGCCAT |  |
| Duet_ N-FLAG_Tde5_R | GGCCGGCCGATATCTCAGATGTAACCTTTGCATTTTGCCC |  |
| pET29b_Tdi5b_C-VSV-G_F | GAGATATACATATGAAGGACATCCTTAAAGATTTTCTAGAAG | For construction of pET29b::Tdi5b_C-VSV-G; pull-down |
| pET29b_Tdi5b_C-VSV-G_R | TGGTGGTGCTCGAGTCATTTTCCTAATCTATTCATTTCAATATCTGTATACAGTTTTTTCCCTTCCGGCTC |  |

**Table S5: AlphaFold3 predictions and confidence metrics**

| **Prediction** | **Reference** | **ipTM** | **pTM** |
| --- | --- | --- | --- |
| Tdi5a | 5B | - | 0.86 |
| Tdi5b | 5B | - | 0.80 |
| *A. jatrophae* immunity | S3A | - | 0.91 |
| *Rhizobium sp.* immunity | S3A | - | 0.87 |
| *A. pedis* immunity | S3A | - | 0.85 |
| *H. huttiense* immunity paralog 1 | 5B, S3A | - | 0.91 |
| *H. huttiense* immunity paralog 2 | S3A | - | 0.92 |
| *H. huttiense* immunity paralog 3 | S3A | - | 0.91 |
| Tde5/Mg^2+^ complex | 2A | 0.97 | 0.94* |
| *H. huttiense* Tde5/immunity paralog 1 complex | S3B | 0.94 | 0.92* |
| Tde5/Tdi5a/Tdi5b complex | 4D, S2D | 0.91 | 0.90* |

*pTM values are derived from truncated models corresponding to the regions shown in the figures, with the disordered N-terminal region of Tde5 omitted in each

**References**

76. Cardona, S. T., and Valvano, M. A. (2005) An expression vector containing a rhamnose-inducible promoter provides tightly regulated gene expression in Burkholderia cenocepacia *Plasmid* **54**, 219–228

77. Silverman, J. M., Agnello, D. M., Zheng, H., Andrews, B. T., Li, M., Catalano, C. E. *et al.* (2013) Haemolysin coregulated protein is an exported receptor and chaperone of type VI secretion substrates *Mol Cell* **51**, 584–593
